# The Functions and Mechanisms of PR-DUB in Malignancy

**DOI:** 10.3389/fmolb.2021.657150

**Published:** 2021-03-12

**Authors:** Lei Cao, Rui Li, Xudong Wu

**Affiliations:** ^1^State Key Laboratory of Experimental Hematology, The Province and Ministry Co-sponsored Collaborative Innovation Center for Medical Epigenetics, Cancer Institute and Hospital, Department of Cell Biology, School of Basic Medical Sciences, Tianjin Medical University, Tianjin, China; ^2^Department of Neurosurgery, Tianjin Medical University General Hospital, Tianjin, China

**Keywords:** histone modification, polycomb, H2AK119ub1, myeloid maliganancies, cancers, mutation, transcription regulation

## Abstract

The interplay between cancer genome and deregulated epigenomic control is critical for cancer initiation and progression. *ASXL1* (Additional Sex combs-like 1) is frequently mutated in tumors especially myeloid malignancies. However, there remains a debate whether the mutations are loss or gain-of-function. Mechanistically, ASXL1 forms a complex with BAP1 for the erasure of mono-ubiquitylation at lysine 119 on Histone H2A (H2AK119ub1), a well-known histone mark associated with transcription repression. Unexpectedly, this de-ubiquitylation complex has been genetically defined as a Polycomb Repressive complex though the regulatory mechanisms are elusive. In this review, we will discuss about the functions of ASXL1 in malignancies and reconcile seemingly paradoxical effects of ASXL1 or BAP1 loss on transcription regulation.

## Introduction

Polycomb group (PcG) and trithorax group (TrxG) proteins, are conserved epigenetic regulators from *Drosophila* to mammals and have caught a huge amount of attention in the past decades (Schuettengruber et al., 2017; Kuroda et al., 2020). PcG proteins are critical for controlling cellular identity through maintaining appropriate gene repression profiles by forming Polycomb repressive complexes (PRC). Among the identified subgroups of PRCs, PRC2 mainly acts a histone methyltransferase for H3K27, PRC1, or more specifically non-canonical PRC1 is responsible for H2AK119 monoubiquitylation (H2AK119ub1; Di Croce and Helin, 2013). In contrast, TrxG proteins counteract PcG-mediated gene silencing, mainly by catalyzing methylation at H3K4.

**FIGURE 1:**
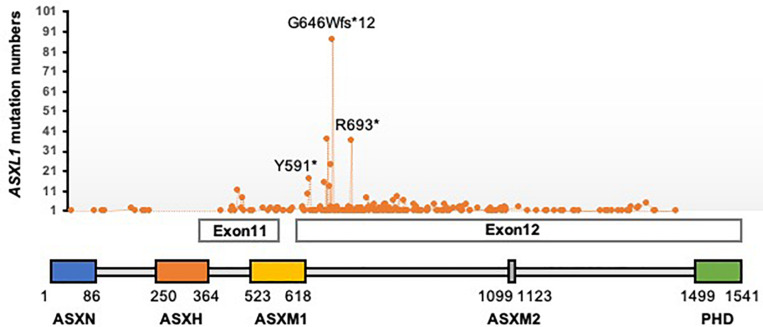
Schematic representation of ASXL1 protein domains and the location of amino acids affected by mutations found in cancers. ^∗^indicates a truncating mutation.

Interestingly, another group of genes with dual functions in the maintenance of both transcriptional activation and repression in *Drosophila* has been categorized as Enhancers of Trithorax and Polycomb (ETP; Gildea et al., 2000). *Asx* was initially identified as one of ETP genes as *Asx*-null mutants exhibit phenotypes of both TrxG and PcG mutants (Sinclair et al., 1998; Milne et al., 1999). Biochemically, Asx interacts with Calypso to form a specific H2AK119 deubiquitylase complex. Loss of Calypso or Asx results in an increase of global H2AK119ub1 levels, however, unexpectedly derepression of PcG target genes. Accordingly this complex was named as Polycomb-repressive deubiquitylase (PR-DUB; Scheuermann et al., 2010). Three mammalian Additional Sex combs-like (ASXL) genes (*ASXL1*, *ASXL2*, and *ASXL3*) have been identified as the orthologs of *Drosophila Asx.* The encoding proteins share common organized regions, an ASXN domain at the *N*-terminal region, an ASX homology (ASXH) domain at the *N*-terminal adjacent region and a plant homeodomain (PHD) domain in the *C*-terminal region. ASXL1 directly binds to BAP1 through the ASXH domain and its adjoining region (also called deubiquitinase adaptor, DEUBAD; Sahtoe et al., 2016; Figure 1). In this mini-review, we focus on ASXL1 and discuss the current functional and mechanistic understanding of mammalian PR-DUB.

## *ASXL1* Mutations in Myeloid Malignancies: Loss or Gain-Of-Functions?

Heterozygous frame-shift or nonsense mutations *of ASXL1*, usually in exons 11 or 12, before the PHD domain (Figure 1), have been the most frequently found in myeloid malignancies, 10–25% in patients with myelodysplastic syndrome (MDS), 40–50% of chronic myelomonocytic leukemia (CMML) patients, 5–11% of patients with primary Acute myeloid leukemia (AML). And the presence of the mutations is associated with adverse outcomes (Gelsi-Boyer et al., 2009, 2010; Boultwood et al., 2010; Chou et al., 2010; Metzeler et al., 2011; Thol et al., 2011; Itzykson et al., 2013). In CMML patients, additional *TET2* mutations were associated with shorter survival in the presence of *ASXL1* mutations (Cui et al., 2015, 2016). In contrast, *ASXL1* mutations are mutually exclusive with *DNMT3A*, *NPM1*, and *SF3B1* mutations (Asada et al., 2019). These correlations should be taken into account for clinical prognosis as well as functional studies.

To date, a variety of mouse models have been taken to clarify roles of ASXL1 in development and diseases. It is interesting to note that mice with Asxl1 loss alone does not develop malignancies. Hematopoietic-specific deletion of *Asxl1* results in progressive leukopenia and dysplasia. The numbers of hematopoietic stem/progenitor cells are increased and their differentiation is impaired (Abdel-Wahab et al., 2013). To better simulate the heterozygous patient-derived mutations, Uni et al. recently generated a mouse model with a heterozygous knock-in of Asxl1^*G*643*fs*^ at the original *Asxl1* locus. And the mutant mice developed phenotypes recapitulating human low-risk MDS and some of the mice developed MDS/myeloproliferative neoplasm-like disease after long latency (Uni et al., 2019). Consistent with the clinical prognosis (Cui et al., 2015, 2016), hematopoietic-specific deletion of both *Asxl1* and *Tet2* leads to a much earlier onset of MDS than the deletion of *Asxl1* or *Tet2* alone. In support, Asxl1 deficiency accompanied with haploinsufficiency of *Nf1* or *KRas* oncogenic mutation accelerates the development of myeloid leukemia in mice (Abdel-Wahab et al., 2012; Zhang et al., 2018). Therefore, these models argue that loss of ASXL1 contributes to the initiation and progression of myeloid malignancies, dependent or independent of its H2AK119 deubiquitylation activity.

Notably the expression of *C*-terminal truncation of ASXL1 has been observed in a few leukemia cell lines (Balasubramani et al., 2015; Xia et al., 2020). Moreover, artificial or transgenic overexpression of ASXL1 *C*-terminal truncated mutants leads to gain of H2AK119 deubiquitylation activity and facilitates myeloid transformation (Inoue et al., 2013; Balasubramani et al., 2015; Asada et al., 2018; Nagase et al., 2018; Yang et al., 2018; Xia et al., 2020). Besides, ASXL1 mutant may synergize with hematopoietically expressed homeobox (HHEX) to promote myeloid transformation. In contrast, CRISPR/Cas9-mediated correction of *ASXL1*-truncated mutation decreases the proliferation of corresponding leukemia cells (Valletta et al., 2015; Takeda et al., 2020). Despite of these gain-of-function evidences for mutant ASXL1, it is yet clear how the globally decreased H2AK119ub1 contributes to malignancy.

## Mechanistic Insights Into H2Ak119ub1 Deregulation in Malignancies

H2AK119ub1 has long been suggested as an important mark for PcG-mediated gene silencing. Hence it is surprising that ASXL1-BAP1 or PR-DUB is genetically defined as a repressive complex. And ASXL1 loss leads to a global reduction of H3K27me3 in certain contexts (Abdel-Wahab et al., 2012; Wang et al., 2014). However, recently, two independent studies have argued that BAP1 is required for the activation of a subset of target genes (Campagne et al., 2019; Kolovos et al., 2020), though direct evidence is lacking whether these activating functions are based on H2AK119 deubiquitylation. And we have demonstrated that ASXL1 is driven to erase H2AK119ub1 for transcription induction. Loss of ASXL1 leads to the retention of H2AK119ub1 at the regulatory loci of *INK4B* and *PTEN* and thereby the inactivation of these two well-known tumor suppressor genes in response to oncogenic signals, supporting ASXL1 as a tumor suppressor (Wu et al., 2015; Cao et al., 2020).

**FIGURE 2:**
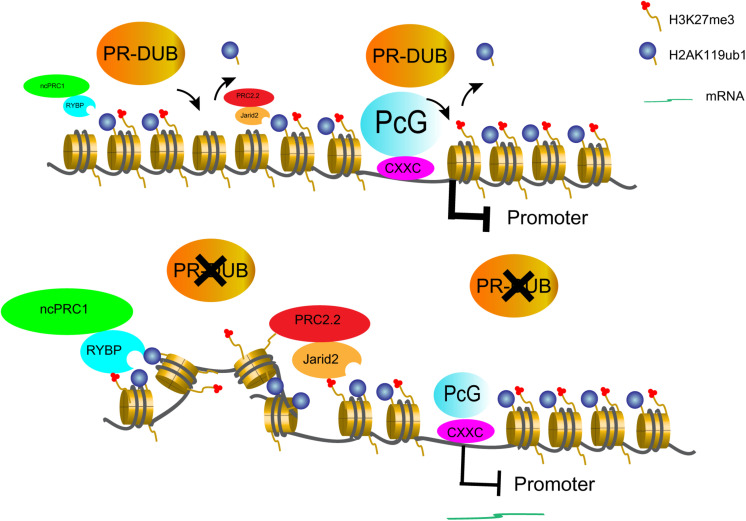
A prospective model to define PR-DUB as a transcription repressive complex. PR-DUB restricts H2AK119ub1 and PcG proteins mainly at promoters. However, upon PR-DUB loss, the gain of H2AK119ub1 at non-promoters attracts PcG proteins away from the target promoters and thereby leads to gene depression.

To resolve the discrepancy, it may require to re-examine the roles of ASXL1 and H2AK119ub1 in transcription regulation. Actually, ASXL1 but not its mutant form has been shown to be incorporated into PRC1 (Uni et al., 2019). In addition, *Asxl1* mutation has been recently reported to correlate with reduced expression of PRC1 members in zebrafish (Fang et al., 2021). Hence, *ASXL1* mutations may lead to loss of PRC1 functions through various mechanisms. As for H2AK119ub1, a diffuse distribution far beyond at promoters has been observed (Loubiere et al., 2020; Tamburri et al., 2020). Notably H2AK119ub1 is recognized by RYBP and JARID2 (Cooper et al., 2016; Zhao et al., 2020), and therefore it facilitates PRC2 recruitment (Kasinath et al., 2021) and the propagation of Polycomb domain (Zhao et al., 2020). Accordingly the low but detectable levels of H2AK119ub1 at non-promoter regions may function as a sponge to attract PRCs. The excessive gain of H2AK119ub1 upon loss of PR-DUB may lead to ineligible PRC relocation from repressive promoters to non-promoter regions and therefore a passive derepression of target genes (Figure 2). The observed decrease of H3K27me3 at PcG-targeted promoters in ASXL1 or BAP1-deficient cells likely follows this model (Abdel-Wahab et al., 2012; LaFave et al., 2015). Furthermore, non-canonical PRC1 and H2AK119ub1 have recently been shown to be associated with active histone marks (Yao et al., 2018; Cohen et al., 2020) and gene responsiveness in *Arabidopsis* (Kralemann et al., 2020; Yin et al., 2021). Considering that ubiquitin is almost half size of core histones and may affect the dynamics of chromatin architecture, we envisage that H2AK119ub1 may confer responsiveness to transcription induction for unknown reasons.

## Discussion

Emerging studies have challenged the traditional viewpoint that chromatin modifications are instructive for transcription regulation (Morgan and Shilatifard, 2020). In this scenario, H2AK119ub1 does not have to determine transcriptional repression though it is associated with inactive genes. Either defining H2AK119ub1 as an active or repressive mark may be over-simplified. Hence as an eraser, PR-DUB removes H2AK119ub1 to reconfigure the chromatin landscape, rather than simply turn on or off gene transcription. It is well recognized that the epigenome function is to allow phenotypic variation in adaptation to the changing environment. *ASXL1* mutations, leading to either gain or loss of H2AK119 deubiquitylation activity and therefore overly permissive or restrictive chromatin landscape, causes improper adaptation to intrinsic or extrinsic signals and malignant transformation. In a broad sense, a proper interpretation of the effects of epigenetic changes on transcription is critical for functional insight into chromatin modifiers in development and diseases settings.

## Author Contributions

LC and XW wrote the manuscript. LC and RL prepare the figures. All authors contributed to the article and approved the submitted version.

## Conflict of Interest

The authors declare that the research was conducted in the absence of any commercial or financial relationships that could be construed as a potential conflict of interest.
